# Evaluating the relationship between ocular blood flow and systemic organ blood flow in hemorrhagic shock using a rabbit model

**DOI:** 10.1038/s41598-024-54467-1

**Published:** 2024-02-14

**Authors:** Kento Watanabe, Tomoaki Shiba, Akira Takahara, Hiroshi Homma, Tetsuya Komatsu, Yusuke Tanino, Yoshinobu Nagasawa, Megumi Aimoto, Yuichi Hori

**Affiliations:** 1 Department of Ophthalmology, Toho University, 6-11-1, Omorinishi, Oota-ku, Tokyo, 143-8541 Japan; 2 Department of Ophthalmology, International University of Health and Welfare Narita Hospital, Chiba, Japan; 3 Department of Pharmacology and Therapeutics, Faculty of Pharmaceutical Sciences, Toho University, Chiba, Japan; 4 Department of Emergency and Critical Care Medicine, Tokyo Medical University, Tokyo, Japan

**Keywords:** Blood flow, Retina

## Abstract

This study aimed to investigate the feasibility of utilizing noninvasive ocular blood flow measurements as potential indicators of systemic circulation in rabbits experiencing hemorrhagic shock. Using Laser speckle flowgraphy, ocular blood flow indices, relative flow volume (RFV), and mean blur rate in the choroidal area (MBR-CH) were assessed in New Zealand White rabbits (n = 10) subjected to controlled blood removal and return. Hemodynamic parameters and biochemical markers were monitored alongside ocular circulation during blood removal and return phases. Additionally, correlations between ocular parameters and systemic indices were examined. The results indicated that RFV and MBR-CH exhibited significant correlations with renal and intestinal blood flows, with stronger correlations observed during blood removal. Additionally, ocular blood flow changes closely mirrored systemic dynamics, suggesting their potential as real-time indicators of shock progression and recovery. These findings indicate that ocular blood flow measurements may serve as real-time indicators of the systemic circulation status during hemorrhagic shock, offering potential insights into shock management and guiding tailored interventions. Thus, noninvasive ocular blood flow evaluation holds promise as an innovative tool for assessing systemic circulation dynamics during hemorrhagic shock.

## Introduction

Hemorrhagic shock is organ damage caused by inadequate blood flow in peripheral tissues, resulting from decreased circulating plasma volume due to hemorrhage. Owing to the difficulty in direct observation of organ blood flow, although a comprehensive evaluation is conducted by combining vital signs, physical findings, and various examinations, no definitive conclusion has been reached regarding the appropriate index for circulatory resuscitation. Optical techniques, such as Near-Infrared Spectroscopy (NIRS) and Raman spectroscopy, are widely used to examine microcirculation in tumors^1^ and peripheral arterial diseases^2^ and to characterize various biological materials^3^. Moreover, in recent years, the evaluation of microcirculation in sublingual^4–8^ and body surface tissue^9–13^ has attracted attention for shock management and is considered a better predictor of prognosis than macro-circulation indicators such as blood pressure.

Retinal vessels are the only vessels that allow direct observation of an organ’s blood supply and have been used for systemic evaluation such as arteriosclerosis for a long time^14–16^. To date, studies have explored the relationship between the ocular blood flow index of fluorescent fundus angiography using contrast media and the Acute Physiology and Chronic Health Evaluation II (APACHE II) score and cardiac index in patients with sepsis who were hospitalized in the ICU^17^. Additionally, animal experiments investigating changes in structural ocular microcirculation during shock have been reported^18^. However, these previous reports had problems of invasiveness due to the contrast media and difficulty in quantification.

Laser speckle flowgraphy (LSFG, Softcare Ltd., Fukuoka, Japan) quantifies ocular blood flow non-invasively and in real time^19,20^ and can solve the problems encountered to date. We have reported that ocular blood flow measured non-invasively using LSFG reflects systemic circulation indices like blood pressure and cardiac output during the process of hemorrhagic shock progression and recovery using New Zealand White rabbits^21^.

In this study, as a follow-up report, we comprehensively examined the relationships between ocular blood flow and organ blood flow in the kidney and intestine using rabbits with hemorrhagic shock in the progressive phase of shock due to blood loss and in the recovery phase due to blood return. We aimed to investigate the extent to which ocular blood flow actually reflects organ blood flow.

## Results

The amount of blood loss associated with the laparotomy was negligible in all cases. Following the commencement of the blood removal procedure, mean arterial blood pressure (MAP), renal blood flow (RBF), intestinal blood flow (IBF), central venous oxygen saturation (ScvO_2_), and LSFG parameters [% relative flow volume (RFV) and % mean blur rate (MBR) in the choroidal area (MBR-CH)] began to immediately decrease and showed a significant decrease at the end of blood removal. All parameters showed improvement to approximately the reference value by the blood return operation. Conversely, lactic acid (Lac) and creatinine began to increase with blood removal, with Lac peaking at the end of blood return and then declining, whereas creatinine continued to increase thereafter. The heart rate (HR) showed an increasing trend with blood removal and a decreasing trend with blood return, but none of the variations were significant.

**Figure 1:**
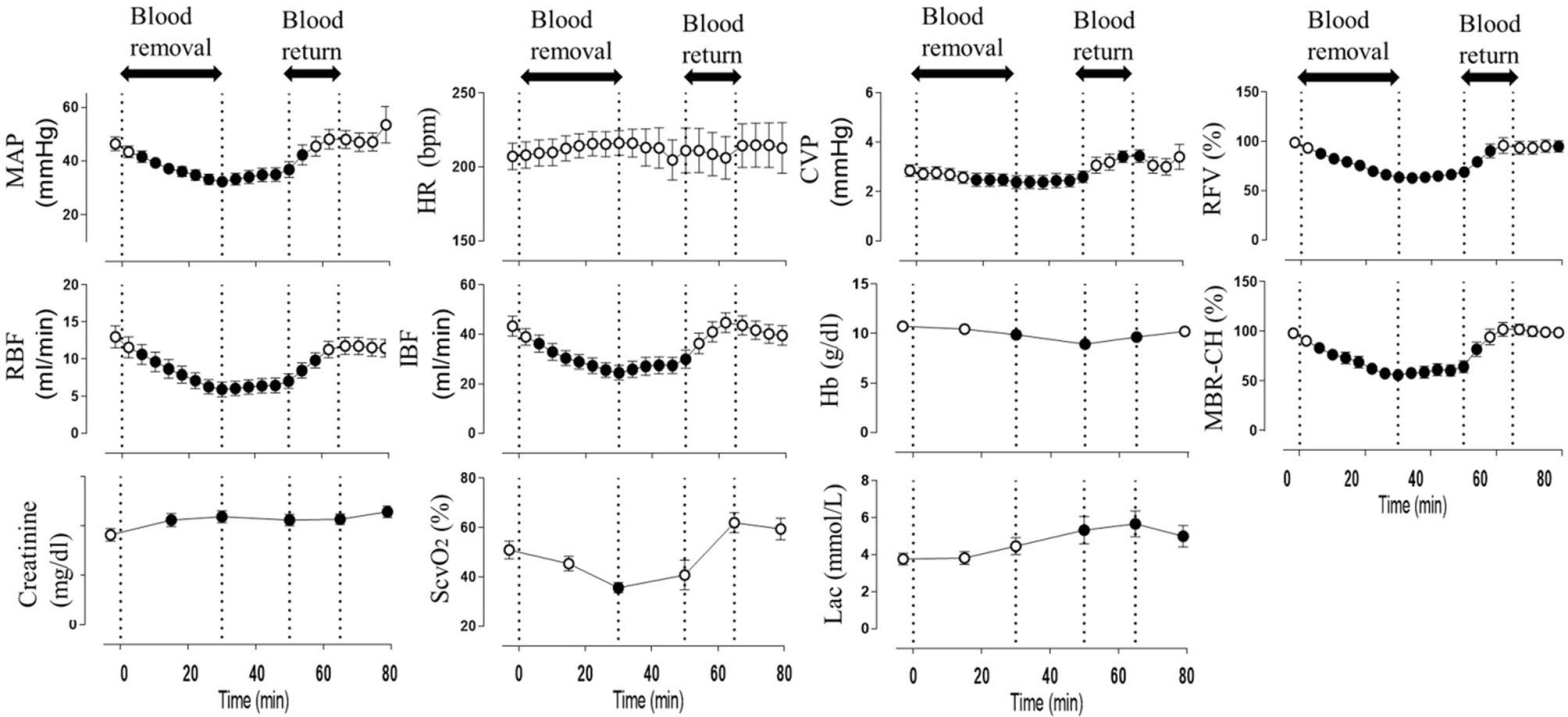
Time courses of the effects of blood removal and blood return on creatinine. *CVP* central venous pressure, *Hb* hemoglobin, *HR* heart rate, *IBF* intestinal blood flow, *Lac* lactic acid, *MAP* mean arterial pressure, *MBR-CH* mean blur rate in the choroidal area, *RBF* renal blood flow, *RFV* relative flow volume, *ScvO*_*2*_ central venous oxygen saturation. Closed symbols: significant difference from the corresponding control value of each parameter indicated by p < 0.05.

**Figure 2:**
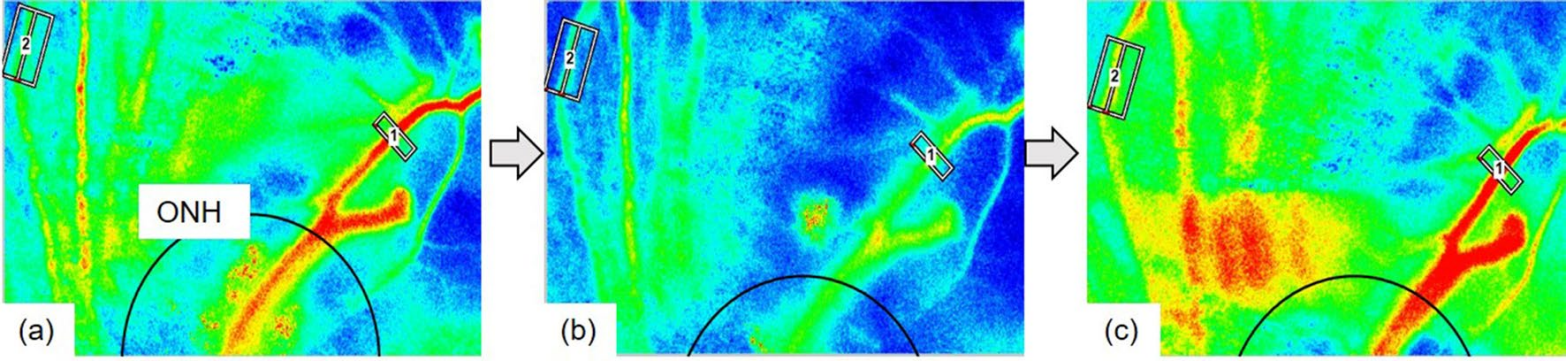
Representative case showing the relative flow volume (RFV) and mean blur rate in the choroidal area (MBR-CH) at the baseline (**a**: RFV = 246, MBR-CH = 8.2), at the end of blood removal (**b**: RFV = 110, MBR-CH = 5.7), and at the blood return phase (**c**: RFV = 321, MBR-CH = 11.1). Rectangles 1 and 2 are placed on the retinal vessel and choroidal area. The circle in the figure indicates the optic nerve head (ONH).

**Figure 3:**
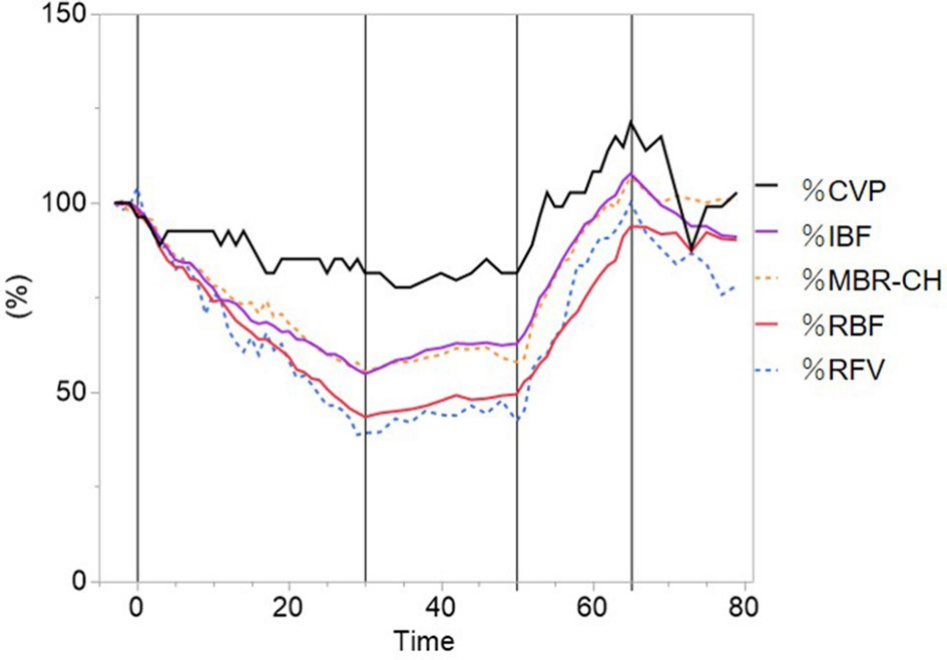
Time courses of the change rate of the central venous pressure (%CVP), intestinal blood flow (%IBF), mean blur rate in the choroidal area (%MBR-CH), renal blood flow (%RBF), and relative flow volume (%RFV) by blood removal and blood return. Change rates were calculated using the following formula: (the values at each measured point/the values at the beginning of the experiment).

The time courses of the changes in the systemic circulation index and LSFG parameters (%RFV and %MBR-CH) are shown in Fig. 1. Figure 2 shows the actual LSFG measurement screens at baseline, at the end of blood removal, and at the end of blood return. The warm color represents high blood flow, and the cold color represents low blood flow. Figure 3 summarizes the time courses of central venous pressure (CVP), RFV, RBF, MBR-CH, and IBF. The RFV, RBF, MBR-CH, and IBF showed similar changes. The change rate of RFV vs. RBF/MBR-CH vs. IBF at the end of blood loss, the beginning of blood return, and the end of blood return, respectively, was 39% vs. 44%/55% vs. 55%, 42% vs. 50%/58% vs. 63%, and 100% vs. 94%/107% vs. 108%. Among them, the change rates in the MBR-CH and IBF at the end of blood removal were significantly equivalent (p = 0.049).

**Figure 4:**
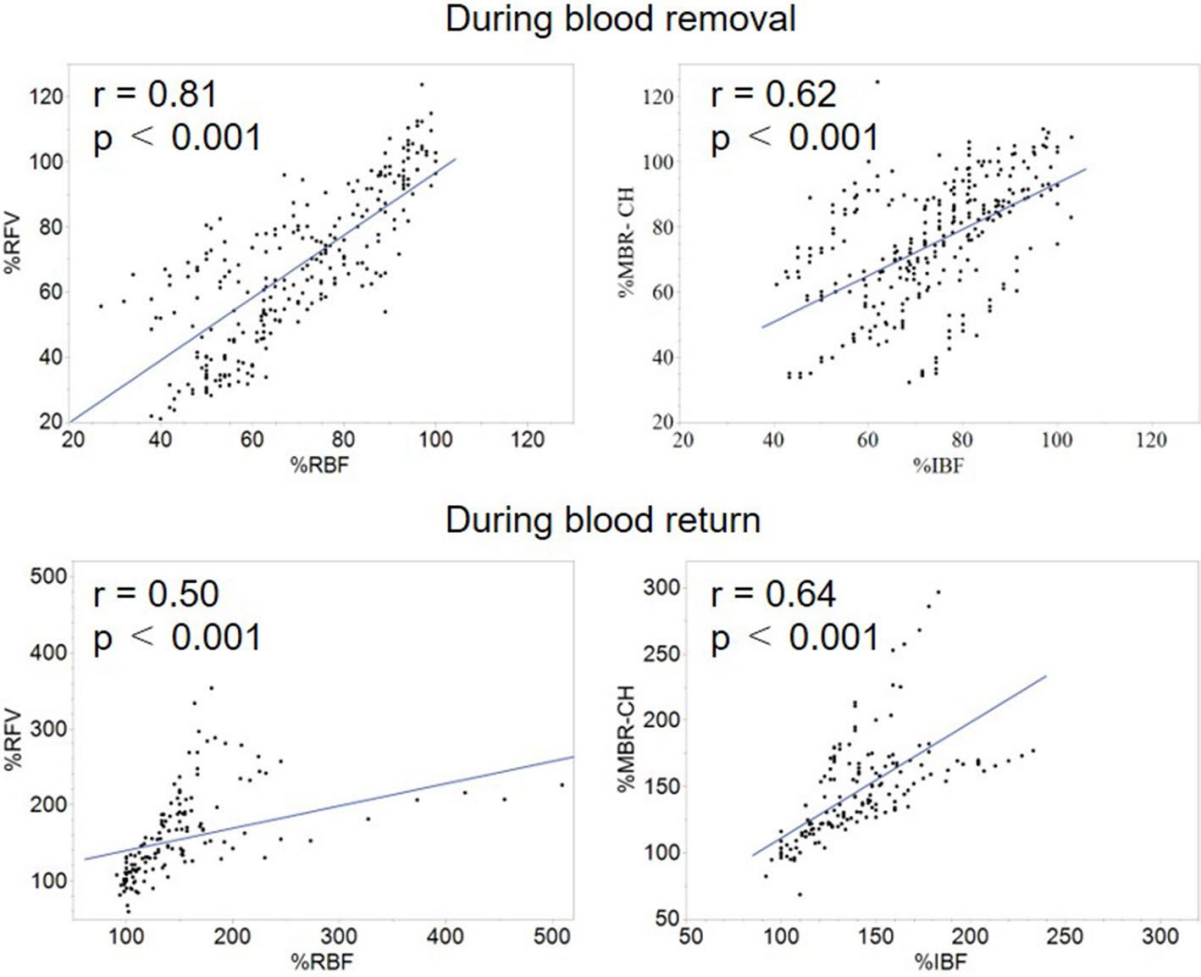
Correlation coefficients between relative flow volume (%RFV) and renal blood flow (%RBF), mean blur rate in the choroidal area (%MBR-CH), and intestinal blood flow (%IBF) during blood removal and blood return. The upper row shows the correlations during the blood removal period, and the lower row shows the correlations during the blood return period. Correlations during the blood removal and return periods were analyzed using change rates, which were calculated using the following formula: (the values at each measured point/the values at the beginning of the experiment and blood return operation).

The correlations coefficients between %RFV, %MBR-CH, %systemic hemodynamics, and laboratory parameters during the blood removal and return periods are presented in Tables 1 and 2. The %RFV and %MBR-CH showed significant positive correlations with %MAP, %RBF, %IBF, and %ScvO_2_. Moreover, %CVP showed positive correlations with %RFV and %MBR-CH only during blood return. %hemoglobin (Hb) exhibited positive correlations with %RFV and %MBR-CH during blood removal and with %MBR-CH during blood return. %Lac showed a significant negative correlation with %MBR-CH during blood removal; however, no significant correlation was seen during the blood return period. Similarly, %creatinine showed significant negative correlations with %RFV and %MBR-CH only during blood removal. Figure 4 shows the graphs of the correlation coefficients between RFV and RBF, MBR-CH, and IBF during blood removal and blood return.

**Table 1:** Correlation coefficients between %RFV, %MBR-CH, and %systemic circulation parameters during blood removal.

Objective variable	%RFV		%MBR-CH	
Explanatory variables	r	p	r	p
% MAP	0.90	< 0.001	0.78	< 0.001
% HR	− 0.39	< 0.001	0.09	0.11
% RBF	0.81	< 0.001	0.78	< 0.001
% IBF	0.75	< 0.001	0.62	< 0.001
% CVP	0.07	0.23	− 0.05	0.36
% ScvO_2_	0.80	< 0.001	0.67	< 0.001
% Lac	− 0.33	0.09	− 0.36	< 0.05
% Hb	0.79	< 0.001	0.72	< 0.001
% creatinine	− 0.86	< 0.001	− 0.61	0.007

A total of 310 data points obtained from 10 rabbits were used to determine the correlation coefficient. Each index is compared by the percentage change from the reference value at the beginning of the experiment. *CVP* central venous pressure, *Hb* hemoglobin, *HR* heart rate, *IBF* intestinal blood flow, *Lac* lactic acid, *MAP* mean arterial pressure, *MBR-CH* mean blur rate in the choroidal area, *RBF* renal blood flow, *RFV* relative flow volume, *ScvO*_*2*_ central venous oxygen saturation.

**Table 2:** Correlation coefficients between %RFV, %MBR-CH, and %systemic circulation parameters during blood return.

Objective variable	%RFV		%MBR CH	
Explanatory variables	r	p	r	p
% MAP	0.77	< 0.001	0.74	< 0.001
% HR	− 0.04	0.64	− 0.07	0.39
% RBF	0.50	< 0.001	0.35	< 0.001
% IBF	0.84	< 0.001	0.64	< 0.001
% CVP	0.65	< 0.001	0.47	< 0.001
% ScvO_2_	0.72	< 0.001	0.84	< 0.001
% Lac	0.05	0.86	0.17	0.49
% Hb	0.37	0.13	0.58	< 0.01
% creatinine	− 0.10	0.78	0.08	0.81

A total of 160 data points obtained from 10 rabbits were used to determine the correlation coefficient. Each index is compared by the percentage change from the reference value at the beginning of the experiment. *CVP* central venous pressure, *Hb* hemoglobin, *HR* heart rate, *IBF* intestinal blood flow, *Lac* lactic acid, *MAP* mean arterial pressure, *MBR-CH* mean blur rate in the choroidal area, *RBF* renal blood flow, *RFV* relative flow volume, *ScvO*_*2*_ central venous oxygen saturation.

## Discussion

During the stages of progression and recovery from hemorrhagic shock, changes in blood pressure, HR, ScvO_2_, Lac, and Hb were consistent with our previous report^21^. Ocular blood flow indices (RFV, MBR-CH) reflected blood pressure and other parameters and decreased and recovered with the progression and recovery process of shock, respectively. In this study, we compared the relationships between ocular, renal, and intestinal blood flows during the progression and recovery phases of shock.

The RFV and MBR-CH during the blood removal period exhibited substantial positive correlations not only with blood pressure, Lac, and ScvO_2_, which are indicators of shock, but also with RBF and IBF. Moreover, the change rates of blood flow at the end of blood removal were RFV, 39% vs. RBF, 44% and MBR-CH, 55% vs. IBF, 55%, showing significant equivalence between MBR-CH and IBF. As shock progresses, ocular blood flow indices correlated with Lac and creatinine levels, which are commonly used biochemical indicators of shock and renal dysfunction. Furthermore, the blood vessels of the eye, referred to as “strain vessels^22^” are believed to have the same structure and role as the kidneys and coronary arteries, which may allow us to predict the degree of renal and intestinal hypoperfusion by observing ocular blood flow during the progression of hemorrhagic shock.

RFV and MBR-CH showed a linear decrease with the progression of shock due to the blood removal maneuver, similar to the RBV and IBF. Although the retinal vessels and kidneys are generally considered autoregulatory vessels and organs^23–25^, respectively, their capacities are limited, and they are less active when blood pressure decreases than when it is elevating^26,27^. Therefore, rapid hemorrhagic shock was thought to cause autoregulation failures and decreases in ocular, renal, and intestinal blood flows.

During the blood return period, RFV and MBR-CH showed significant positive correlations with RBF and IBF; however, the degree of correlations were weaker with RBF than that during the blood removal period. This may be attributed to the difference in the degree of autoregulation by each organ and the intraocular region in response to increased blood flow. However, even during blood return, RFV showed significant correlations with RBF (r = 0.5) and IBF (r = 0.84), suggesting that ocular blood flow measurement may be applied to evaluate the status of systemic organ blood flow, even during the shock recovery process.

Additionally, ocular blood flow was negatively correlated with Lac and creatinine levels during shock progression but not during blood return. This was believed to be due to a time lag in the recovery of Lac and creatinine^28^, which caused a gap in other circulatory and ocular blood flow indices. Therefore, using only biochemical data as indicators of infusion therapy may lead to excessive infusion^29^. However, ocular blood flow measurements can reflect organ blood flow in real time, and we believe that adding information on ocular blood flow to other circulatory indicators would allow for more appropriate infusion therapy without excess or deficiency.

This study had certain limitations. The blood pressure was low, at 46.7 mmHg, at the beginning of the experiment, and invasion by laparotomy could not be ruled out. However, removal of 30 mL of blood, which is about 20% of the total blood volume, caused a 36% decrease in blood pressure and a significant increase in the lactic acid level (from 3.8 to 5.7 mmol/L, p = 0.043) and a significant decrease in ScvO_2_ (from 50.9 to 35.5%, p = 0.018), indicating that the blood removal operation advanced the shock state in this experiment. Moreover, similar to blood pressure, the possibility that general anesthesia affects ocular autoregulation cannot be ruled out. However, in a report using the same system^30,31^, an autoregulatory-like response was observed at similar blood pressures. Therefore, despite the constraints of limited conditions, we considered it would be possible to examine the relationship between ocular and organ blood flows.

Also, note that the absolute blood flow measurement has not yet been established with LSFG, and quantification is performed using arbitrary units^32^. However, conjunctival video capillaroscopy and laser doppler velocimetry can quantify absolute blood flow; thus, we will consider measurements using these methods and comparisons with LSFG in the future.

Moreover, we maintained the IOP at 10 mmHg in this study. However, ocular perfusion pressure is defined as 2/3 MAP—IOP^33^, and rapid blood pressure reportedly changes the influence on IOP^34^. Therefore, the IOP recorded in this study does not represent the physiological situation, and we cannot rule out the possibility that this may affect ocular blood flow measurements.

Generally, the Hb concentration does not change immediately after the start of blood removal. However, in this case, the Hb concentration was considered to have decreased immediately after the start of blood removal because of the continuous saline infusion during the blood removal operation. In addition, it was reported that the hematocrit level had almost no effect on the MBR within the physiological range^35^.

In this study, LSFG was used to assess microcirculation, looking at a different level of circulation, as opposed to larger feeding vessels for the kidney and intestine. However, since this study also found correlations between ocular circulation and biochemical data such as creatine and lactic acid, we believe that ocular circulation may also reflect microcirculation disorders in the kidneys and intestinal tract to some extent. Thus, in the future, we think it will be necessary to investigate using blood vessels at the same level in the eye and organs.

Clinical studies have reported associations between ocular circulation and severity scores^17^ and mortality^36^, which may support the application of the measurement of the blood flow in the eye, a peripheral organ, to the evaluation of systemic circulation in humans. In addition, the treatment of hemorrhagic shock is centered on fluid and blood transfusion therapy. Although various evaluation methods have been proposed to date to determine the appropriate dosage, none of them directly observe organ blood flow, which is the goal of treatment of hemorrhagic shock. In this study, ocular blood flow was shown to reflect blood flow to major organs throughout the body, and its measurement may be quantified non-invasively at the bedside in real time. These findings suggest that the evaluation of ocular blood flow warrants future clinical research as a new method for evaluating circulation that may solve problems such as excessive transfusion.

In conclusion, noninvasive ocular blood flow measurement using LSFG reflected renal and intestinal blood flow in rabbits with hemorrhagic shock.

## Methods

### Animals and measurements

We used 10 male New Zealand White rabbits (16–18 weeks old, weight 2.90–3.40 kg, median 3.18 kg) housed in the same environment (specific pathogen free). The experiments were performed under general anesthesia. Anesthesia was induced using ketamine (35 mg/kg, i.m., Daiichi Sankyo Propharma, Tokyo, Japan) and xylazine (5 mg/kg, i.m., Bayer Japan, Osaka, Japan) and maintained using isoflurane (end-tidal concentration of 1.5%, Pfizer Japan, Tokyo, Japan). After tracheostomy, the rabbits were mechanically ventilated (FiO_2_ = 1.0, tidal volume = 6 mL/kg, 40 strokes/min; SN-480-5^®^, Shinano, Tokyo, Japan). Saline (15 mL/h) and rocuronium (0.6 mg/kg/h; Fuji Pharma, Toyama, Japan) were continuously administered via a left ear vein. The rabbits’ body temperature was maintained at 37 °C with a heating pad.

All animal experiments were approved by Toho University Laboratory Animal Research (#19-53-358, #20-54-358) and performed in accordance with the Guiding Principles for the Care and Use of Laboratory Animals approved by The Japanese Pharmacological Society. This study also complies with the ARRIVE guidelines.

**Figure 5:**
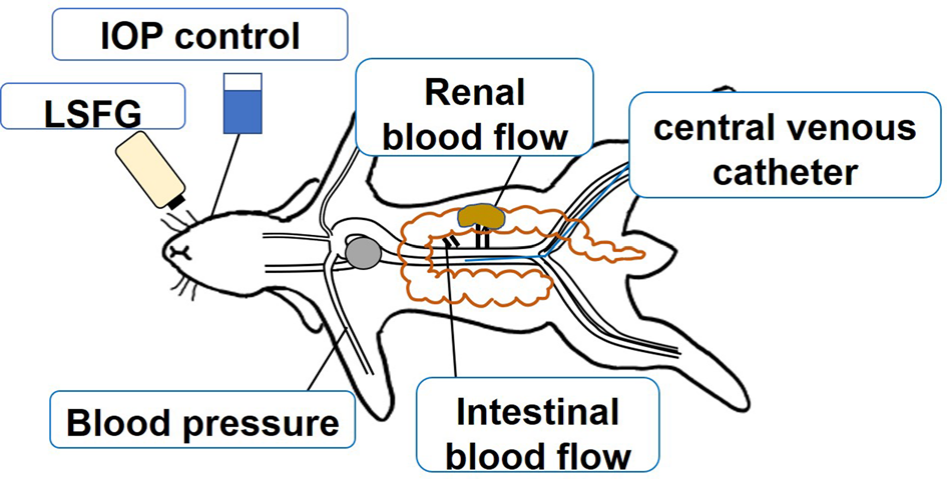
Schematic diagram of the experiment. Blood pressure was measured by placing an arterial catheter in the right upper-arm artery. Intestinal blood flow was measured in the superior mesenteric artery. Renal blood flow was measured in the left renal artery. A central venous catheter was inserted through the left femoral vein, and the tip was placed in the inferior vena cava. LSFG (laser speckle flowgraphy) was performed in the left eye. To maintain the intraocular pressure (IOP), a 25-ga. infusion cannula was inserted into the vitreous cavity.

**Figure 6:**
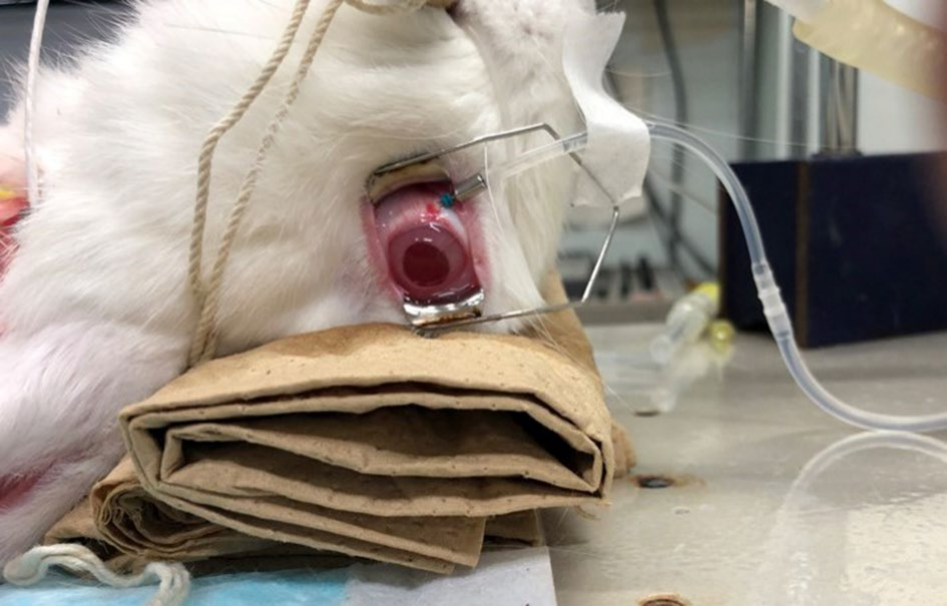
A photograph of LSFG measurement. The rabbits were placed in the supine position. We measured the rabbit’s left eye, held open using an eyelid device. To maintain the intraocular pressure (IOP), a 25-ga. infusion cannula was inserted into the vitreous cavity.

A heparinized catheter was inserted into the right brachial artery for the continuous measurement of blood pressure using transducer. For blood removal and central venous pressure measurement, a catheter was inserted into the femoral vein, and the tip of the catheter was placed in the inferior vena cava. MAP, HR, CVP were recorded using a PowerLab system^®^ (AD Instruments, Sydney, Australia). To measure IBF (mL/min) and RBF (mL/min), a blood flow meter was attached to the superior mesenteric artery exposed by laparotomy, and the renal artery was exposed from the retroperitoneum. The IBF and RBF were measured using an ultrasonic blood flowmeter (TS420^®^, Transonic Systems, Ithaca, NY, USA). Ultrasonic flow meters utilize the ultrasound transit time method. Blood flow rate can be estimated without measuring vessel diameter by integrating the velocity-chord products of each ultrasonic beam obtained using the differences in the propagation time of the forward and reverse ultrasound waves relative to the direction of the blood flow, which are obtained using two transducers and an acoustic reflector^37^. The abdomen was closed with sutures to reduce insensible water loss. A plastic cover protected the blood flow meter from shifting. Figure 5 illustrates the apparatus used to measure systemic hemodynamics and ocular microcirculation in rabbits. Moreover, Fig. 6 is a photograph of actual LSFG measurement scene.

### Experimental protocol

After confirming that the rabbits’ systemic hemodynamics and ocular circulation state were stable for approximately 30 min, the experiments were initiated. To induce hemorrhagic shock, blood was continuously removed from the central venous catheter (4-Fr. ga.) at the rate of 1 mL/min for 30 min. At the end of the 30-min blood removal period, an observation was conducted for 20 min, and heparinized blood was returned via the left auricular vein for 15 min (2 mL/min). After returning 30 mL of blood, another observation was conducted for 15 min. After completing the experiments, the rabbits were sacrificed using a rapid intravenous injection of saturated potassium chloride. Systemic hemodynamic parameters were measured simultaneously every 2 min during the observation periods and every 1 min during the blood removal and return periods. In addition, Hb (g/dL), Lac (mmol/L), ScvO_2_ (%), and creatinine (mg/dL) were measured using a blood analyzer (i-STAT 1 Analyzer^®^, Abbott, Chicago, IL). Blood samples (0.5 mL/time) were collected at six time points: at the beginning of blood removal, 15 min after the start of blood removal, the end of blood removal, the beginning of blood return, the end of blood return, and the end of the experiment.

### Ocular blood flow evaluation

LSFG measurements were determined from fundus images as previously described^19,21,38,39^. Their mechanism are based on changes in the speckle pattern of the laser light reflected from the fundus of the eye^40^. These changes in the speckle pattern depend on the flow velocity of erythrocytes in the vessels and can be used to determine the MBR, which is an indicator of ocular blood flow^38,41^. The MBR reflects blood flow velocity, but it also correlates linearly with capillary blood flow volume^38,42^. The RFV derived from LSFG reflects blood flow velocity and volume in the retinal vessels^32,43^.

The MBR images were obtained using an LSFG-MRC^®^ device (Softcare Ltd., Fukuoka, Japan). LSFG showed good reproducibility^44^. Ocular blood flows are divided into retinal circulation from the central retinal artery and choroidal circulation from the posterior ciliary artery. We measured the RFV in the retinal vessel and the mean MBR-CH in choroidal area. LSFG can place a band in any region to obtain the RFV and MBR within that region. To measure RFV, the band was placed in a straight section just outside the optic head and to measure MBR-CH, the band was placed in an area as far away from the retinal vessels as possible within the angle of view. In each experiment, rectangular bands were stored in the software program, and the same rectangular bands were used for all analyses. The RFV and MBR-CH were calculated using LSFG Analyzer software (ver. 3.8.04. Softcare, Co., Ltd). RFV and MBR-CH were measured simultaneously as the systemic circulation indices every 2 min during the observation period and every 1 min during the blood removal and blood return periods.

Mydriasis was induced by tropicamide eye drops (Santen Pharmaceutical Co., Osaka, Japan). To prevent the corneal surface from drying, saline-containing hydroxyethyl cellulose gel (Senju Pharmaceutical Co. Ltd., Osaka, Japan) was dropped appropriately. Only the left eye was used in all experiments. We kept the IOP at 10 mmHg during each experiment using the below methods. A 25 -ga. infusion cannula was inserted into the vitreous cavity of the rabbit through the pars plana. This infusion cannula was connected to a bottle of intraocular irrigating solution (BSS Plus^®^; Alcon Japan, Tokyo). The IOP was maintained at 10 mmHg by changing the height of the bottle. The IOP was confirmed by an ICARE Tonolab Tonometer^®^ (Revenio Group, Helsinki, Finland) at the start and end of the experiment.

### Statistical analyses

RFV and MBR-CH were evaluated based on the rate of change from the baseline value before blood removal (%RFV, %MBR-CH) because they are not established to compare absolute values between individuals due to differences in blood vessel diameter, blood vessel wall thickness, and race, among others^45^. The time courses of the changes in systematic and ocular hemodynamic parameters during the experiment were analyzed using repeated-measures ANOVA and Dunnett’s tests as post hoc tests. The change rates were calculated using the following formula: (the values at each measured point/the values at the beginning of the experiment). To examine the equivalence between RBF and RFV and IBF and MBR-CH at the end of blood removal, we used two one-sided tests with a 10% margin of equivalence. Correlation coefficients between the change rates (%) of systemic hemodynamics (%MAP, %HR, %RBF, %IBF, %CVP), %laboratory parameters (%ScvO_2_, %Lac, %Hb, %creatinine), and LSFG parameters (%RFV and % MBR-CH) were analyzed using Pearson’s coefficient during blood removal and blood return. Correlations during the blood removal and return periods were analyzed using change rates, which were calculated using the following formula: (the values at each measured point/the values at the beginning of the experiment and blood return operation). Continuous variables are presented as mean ± standard error. Statistical significance was set at p < 0.05. The JMP-10.0 program^®^ (SAS, Cary, NC, USA) was used for the statistical analyses.

## Data Availability

The datasets generated and/or analyzed during the current study are available from the corresponding author on reasonable request.
